# Acute HIV infection (AHI) in a specialized clinical setting: case-finding, description of virological, epidemiological and clinical characteristics

**DOI:** 10.7448/IAS.17.4.19676

**Published:** 2014-11-02

**Authors:** Adriana Ammassari, Isabella Abbate, Nicoletta Orchi, Carmela Pinnetti, Gabriella Rozera, Raffaella Libertone, Paola Pierro, Federico Martini, Vincenzo Puro, Enrico Girardi, Andrea Antinori, Maria Rosaria Capobianchi

**Affiliations:** 1 Clinical Department, National Institute for Infectious Disease, Rome, Italy; 2 Laboratory of Virology, National Institute for Infectious Disease, Rome, Italy; 3 Epidemiology Department, National Institute for Infectious Disease, Rome, Italy; 4 Laboratory of Immunology, National Institute for Infectious Disease, Rome, Italy

## Abstract

**Introduction:**

Diagnosis of HIV infection during early stages is mandatory to catch up with the challenge of limiting HIV viral replication and reservoirs formation, as well as decreasing HIV transmissions by immediate cART initiation.

**Objectives:**

Aims were to describe (a) virological characteristics of AHI identified, (b) epidemiological and clinical factors associated with being diagnosed with AHI.

**Methods:**

Cross-sectional, retrospective study. All individuals diagnosed with AHI according to Fiebig's staging between Jan 2013 and Mar 2014 at the INMI “L. Spallanzani” were included. Serum samples reactive to a fourth generation HIV-1/2 assay (Architect HIV Ag/Ab Combo, Abbott) were retested with another fourth generation assay (VIDAS DUO HIV Ultra, Biomérieux) and underwent confirmation with HIV-1 WB (New Lav I Bio-Rad) and/or with Geenius confirmatory assay (Bio-Rad). WHO criteria (two *env* products reactivity) were used to establish positivity of confirmatory assays. In case of clinically suspected AHI, HIV-1 RNA (Real time, Abbott) and p24 assay (VIDAS HIV P24 Bio-Rad) were also performed. Avidity test was carried out, on confirmed positive samples lacking p31 reactivity, to discriminate between recent (true Fiebig V phase) and late infections; to avoid possible misclassifications, clinical data were also used. Demographic, epidemiological, clinical and laboratory data are routinely, and anonymously recorded in the SENDIH and SIREA studies.

**Results:**

During the study period, we observed 483 newly HIV diagnosed individuals, of whom 40 were identified as AHI (8.3%). Fiebig classification showed: 7 stage II/III, 13 stage IV, 20 stage V. Demographic, epidemiological, and clinical characteristics of patients are shown in the Table. Overall, the study population had a median S/Co ratio at fourth generation EIA (Architect) of 49.50 (IQR, 23.54–98.05): values were significantly lower in Fiebig II-IV than in Fiebig V (38.68 [IQR, 20.08–54.84] vs 75.72 [IQR, 42.66–249.80], p=0.01). Overall, median HIV-1 RNA was 5.44 log copies/mL (IQR, 4.29–6.18) and the value observed in Fiebig phase II-IV was higher than that found in Fiebig stage V (6.10 [IQR, 5.49–7.00] vs 4.69 [3.71–5.44], p<0.001). Median CD4+ cell count was 596/mmc (IQR, 410–737). cART was started in 26 patients: TDF/FTC/DRV/r/RAL=18; TDF/FTC/DRV/r=2; TDF/FTC/ATV/r=2; TDF+FTC+EFV=2; TDF/FTC/RAL=1; DRV/r+RAL=1.

**Conclusions:**

Integration of careful epidemiological investigation, partner notification, and technical advances in virological testing are key elements in AHI case-finding. Significant differences were found between Fiebig stages II–IV and Fiebig V with regard to virological exams.

**Table 1:** Clinical characteristics and virological exams according to different Fiebig stage

	Fiebig classification		
	
	Stage II/III (n=7)	Stage IV (n=13)	Stage V (n=20)
Male gender, n (%)	7 (100)	13 (100)	19 (95)
Median age, y (IQR)	37 (25–42)	33 (30–46)	34 (29–47)
Reason for testing, n (%) (missing = 4)			
- Symptoms	6 (86)	8 (62)	7 (35)
- Periodic testing	1914)	4 (32)	12 (60)
- Other	0	1 (7.7)	1 (5)
Known HIV + sexual partner, n (%)	0	3 (23)	1
HIV transmission risk, n (%) (missing = 4)			
- MSM	5 (83)	11 (100)	16 (84)
- Heterosexual contact	1 (17)	0	3 (16)
Negative HIV test in past 5 y (missing = 11)	3 (75)	7 (88)	11 (65)
Symptomatic AHI, n (%)	7 (100)	11 (85)	10 (50)
Site of testing, n (%)			
- Outpatient clinic	5 (71)	10 (100)	18 (90)
- Inpatient	2 (29)	0	2 (10)
Virological exams			
4th gen EIA S/Co ratio, median (IQR)	13.35 (4.13–63.10)	40.20 (31.16–59.44)	75.72 (42.66–249.8)
HIV-1 RNA log copies/mL, median (IQR)	7.00 (5.96–7.00)	5.71 (4.71–6.78)	4.69 (3.71–5.44)
Immunological exams			
CD4 + /mmc, median (IQR)	556 (366–696)	535 (397–701)	669 (475–744)

